# 

**Published:** 1859-07

**Authors:** 


### LIST OF COLLABORATORS.

S. G. Armor, M.D., late Professor of Pathology and Clinical Medicine, Missouri Med. College.
John L. Atlee, M.D., late President Pennsylvania State Medical Society.
Walter F. Atlee, M.D., Philadelphia.
Robert G. Barclay, M.D., Jerusalem, Syria.
John Bell, M.D., Philadelphia, formerly Professor of Medicine, Medical College of Ohio.
T. S. Bell, M.D., Professor of Medicine, University of Louisville.
S. M. Bemiss, M.D., Professor of Clinical Medicine, University of Louisville.
L. P. Blackburn, M.D., New Orleans, La.
George C. Blackie, M.D., Professor of Botany, University of Nashville.
G. C. Blackman, M.D., Professor of Surgery, Medical College of Ohio.
J. L. Bobbs, M.D., formerly Professor of Anatomy, Indianapolis.
W. S. Bowen, M.D., New York.
N. Bozeman, M.D., Montgomery, Ala.
J. T. Bradford, M.D., Augusta, Ky.
John H. Brinton, M.D., Lecturer on Operative Surgery, Philadelphia.
W. S. Chipley, M.D., Professor of Medicine, Transylvania University.
A. Clapp, M.D., New Albany, Ia.
D. B. Cliffe, M.D., Franklin, Tenn.
J. Da Costa, M.D., Lecturer on the Practice of Medicine at the Philad. Med. Association.
Samuel H. Dickson, M.D., Professor of Medicine, Jefferson Medical College.
M. Dupierris, M.D., Havana, Cuba.
Richard J. Dunglison, M.D., Philadelphia.
W. F. Edgar, M.D., United States Army.
S. C. Farrar, M.D., Jackson, Miss.
C. S. Fenner, M.D., Memphis, Tenn.
Emil Fischer, M.D., Philadelphia.
Austin Flint, M.D., Professor of Clinical Medicine, Auscultation and Percussion, New Orleans School of Medicine.
Charles Frick, M.D., Baltimore.
W. Y. Gadbury, M.D., Benton, Miss.
O. C. Gibbs, M.D., Chautauque County, New York.
Dr. J. P. Hall, Glasgow, Ky.
William A. Hammond, M.D., United States Army.

JOHN HARDIN, M.D., Professor of Obstetrics, Kentucky School of Medicine, Louisville.

EDWARD HARTSHORNE, M.D., One of the Surgeons to Wills Hospital for Diseases of the Eye, Philadelphia.

E. B. HASKINS, M.D., Professor of Chemistry, Shelby Medical College, Nashville, Tenn.

C. A. HENTZ, M.D., Marianna, Florida.

ADDINELL HEWSON, M.D., One of the Surgeons to Wills Hospital for Diseases of the Eye, Philadelphia.

SAMUEL L. HOLLINGSWORTH, M.D., late Editor of the Medical Examiner, Philadelphia.

WILSON JEWELL, M.D., Philadelphia, President of the Quarantine and Sanitary Convention.

WM. V. KEATING, M.D., Lecturer on Obstetrics and the Diseases of Women, at the Philadelphia Medical Association.

JOHN G. KERR, M.D., Canton, China.

G. A. KUNKLER, M.D., Madison, Ia.

BEN. LOGAN, M.D., Shreveport, La.

A. LOPEZ, M.D., Mobile, Ala., formerly President of the Alabama State Medical Society.

J. AITKEN MEIGS, M.D., Professor of the Institutes of Medicine in the Medical Department of Pennsylvania College.

S. WEIR MITCHELL, M.D., Lecturer on Physiology at the Philad. Med. Association.

GEORGE R. MOREHOUSE, M.D., Philadelphia.

B. I. RAPHAEL, M.D., New York.

J. C. REEVE, M.D., Dayton, Ohio.

L. C. RIVES, M.D., formerly Professor of Midwifery, Medical College of Ohio.

J. LAWRENCE SMITH, M.D., Professor of Chemistry, University of Louisville.

W. C. SNEED, M.D., Frankfort, Ky., late President Kentucky State Medical Society.

C. H. SPILMAN, M.D., Harrodsburg, Ky., late President of Kentucky State Medical Society.

GEO. SUTTON, M.D., Aurora, Ia.

W. L. SUTTON, M.D., Georgetown, Ky., formerly President Kentucky State Medical Society.

HORATIO R. STORER, M.D., formerly one of the Physicians to the Boston Lying-in Hospital, Boston, Mass.

S. THOMPSON, M.D., Albion, Ill., late President of the Illinois State Medical Society.

E. WILLIAMS, M.D., Cincinnati, Ohio.

### Bi-Monthly Bibliographical Record.

### AMERICAN.

Elements of Medicine: being a compendious View of Pathology and Therapeutics; or the History and Treatment of Diseases. By Samuel Henry Dickson, M.D., etc. 8vo., pp. 768. Philadelphia: Blanchard & Lea, 1859. (From the publishers.)

A Treatise on Baths; including Cold, Sea, Warm, Hot, Vapour, Gas, and Mud Baths; also, on Hydropathy, and Pulmonary Inhalation; with a description of Bathing in Ancient and Modern Times. By John Bell, M.D., etc. Second edition, 12mo., pp. 658. Philadelphia: Lindsay & Blakiston, 1859. (From the publishers.)

A Treatise on Gonorrhœa and Syphilis. By Silas Durkee, M.D., etc. With eight colored plates. 8vo., pp. 442. Boston: John P. Jewett & Co., 1859. (From the publishers.)

Observations on the Treatment of Fractures of the Femur; with a New Apparatus, and Report of Seventeen Cases. By T. H. Hobart Burge, M.D., etc., and W. T. Burge, M.D. 12mo., pp. 45. Brooklyn, 1859. (From the authors.)

Transactions of the New Jersey State Medical Society, for 1859. 8vo., pp. 95. Newark, 1859.

New Surgical Treatment for Malformations of the Urinary Bladder. By Daniel Ayres, M.D., etc. Reprint. 8vo., pp. 14. New York, 1859. (From the author.)

Forty-second Annual Report of the State of the Asylum for the Relief of Persons deprived of the Use of their Reason. 8vo., pp. 32. Philadelphia, 1859.

The Progress and Spirit of Medical Science. An Anniversary Discourse, delivered before the New York Academy of Medicine, November 25, 1858. By E. R. Peaslee, M.D., etc. 8vo., pp. 104. New York, 1859. (From the author.)

### FOREIGN.

Anatomy, Descriptive and Surgical. By Henry Gray, F.R.S. Royal 8vo., pp. 754. Philadelphia: Blanchard & Lea, 1859. (From the publishers.)

A Practical Treatise on the Diseases of Infancy and Childhood. By T. H. Tanner, M.D., F.L.S. 12mo., pp. 464. Philadelphia: Lindsay & Blakiston, 1859. (From the publishers.)

A Manual of Elementary Chemistry, Theoretical and Practical. By George Fownes, F.R.S., etc. Edited by Robert Bridges, M.D., etc. 12mo., pp. 600. Philadelphia: Blanchard & Lea, 1859. (From the publishers.)

Defects of Sight and Hearing; their Nature, Causes, Prevention, and General Management. By T. Wharton Jones, F.R.S., etc. Edited by Laurence Turnbull, M.D., etc. 12mo., pp. 247. Philadelphia: Price & Co., 1859. (From Lindsay & Blakiston.)

On the Organs of Vision; their Anatomy and Physiology. By Thomas Nunnely, F.R.C.S.E., etc. 8vo., pp. 373. London: Churchill, 1858.

Practical Observations on the Operations for Strangulated Hernia. By T. H. James, F.R.C.S., etc. pp. 95. London: Churchill, 1859.

A Guide to the Practical Study of Diseases of the Eye. By James Dixon. Second edition, post 8vo. London: Churchill, 1859.

A Treatise upon Penetrating Wounds of the Chest. By Patrick Fraser, M.D., etc. 8vo., pp. 140 London: Churchill, 1859.

The Causation and Prevention of Disease. By John Parkin, M.D., etc. 8vo. London: Churchill, 1859.

On the Hygienic Management of Infants and Children. By T. Herbert Baker, M.D., etc. 8vo., pp. 120. London: Churchill, 1859.

Thirteen Engravings of the Ganglia and Nerves of the Uterus and Heart; for the use of Students in Anatomy and Physiology. By Robert Lee, M.D., F.R.S. Royal 4to. London: Churchill, 1859.

The Diseases of the Stomach; with an Introduction on its Anatomy and Physiology; being Lectures delivered at St. Thomas's Hospital. By William Brinton, M.D. 8vo., pp. 406. London: Churchill, 1859.

On Poisons, in Relation to Medical Jurisprudence and Toxicology. By Alfred S. Taylor, M.D., etc. Fcap. 8vo. Second edition. London: Churchill, 1859.

A Treatise on Medical Electricity, Theoretical and Practical. By T. Althaus, M.D. 8vo., pp. 370. London: Trübner & Co., 1859.

On the Influence of Variations of Electric Tension, as a Remote Cause of Epidemic and other Diseases. By William Craig. 8vo. London: Churchill, 1859.

